# Health-Related Quality of Life in severely injured patients in Finland: an observational cohort study of 325 patients with 1-year follow-up

**DOI:** 10.1186/s13049-024-01216-y

**Published:** 2024-05-15

**Authors:** Antti Riuttanen, Vilma Brand, Jarkko Jokihaara, Tuomas T. Huttunen, Ville M. Mattila

**Affiliations:** 1 Department of Orthopaedics, Tampere University Hospital, Tampere, Finland; 2 Tampere University, Faculty of Medicine and Health Technology Tampere, Tampere, Finland; 3 Department of Orthopaedics, Faculty of Medicine and Health Technology, Tampere University, Tampere University Hospital, Tampere, Finland; 4 Department of Anaesthesia and Intensive Care Medicine, Heart Hospital, Tampere University Hospital, Tampere, Finland

## Abstract

**Background:**

Major trauma has a significant effect on Health-Related Quality of Life (HR-QoL). It is unclear, however, which factors most affect HR-QoL. This study aims to evaluate HR-QoL after severe injury in Finland and determine how different injury patterns and patient-related factors, such as level of education and socioeconomic group, are associated with HR-QoL. We also assess how well different injury scoring systems associate with HR-QoL.

**Methods:**

We retrospectively analyzed 325 severely injured trauma patients (aged ≥ 18 years, New Injury Severity Score, (NISS) ≥ 16, and alive at 1 year after injury) treated in the Intensive Care Unit (ICU) or High Dependence Unit (HDU) of Tampere University Hospital (TAUH) from 2013 through 2016. HR-QoL was assessed with the EQ-5D-3L questionnaire completed during ICU stay and 1 year after injury. HR-QOL index values and reported problems were further compared with Finnish population norms.

**Results:**

The severity of the injury (measured by ISS and NISS) had no significant association with the decrease in HR-QoL. Length of ICU stay had a weak negative correlation with post-injury HR-QoL and a weak positive correlation with the change in HR-QoL. The largest mean decrease in HR-QoL occurred in patients with spinal cord injury (Spine AIS ≥ 4) (-0.338 (SD 0.136)), spine injury in general (Spine AIS ≥ 2 (-0.201 (SD 0.279)), and a lower level of education (-0.157 (SD 0.231)). Patient’s age, sex, or socioeconomic status did not seem to associate with smaller or greater changes in HR-QoL.

**Conclusions:**

After serious injury, many patients have permanent disabilities which reduce HR-QoL. Injury scoring systems intended for assessing the risk for death did not seem to associate with HR-QoL and are not, therefore, a meaningful way to predict the future HR-QoL of a severely injured patient. Recovery from the injury seems to be weaker in poorer educated patients and patients with spinal cord injury, and these patients may benefit from targeted additional measures. Although there were significant differences in baseline HR-QoL levels between different socioeconomic groups, recovery from injury appears to be similar, which is likely due to equal access to high-quality trauma care.

## Introduction

Traumatic injuries are a major cause of morbidity and mortality worldwide, affecting individuals across a wide range of age groups [1]. With advances in trauma care and a reduction in trauma mortality, there is a growing recognition of the need to assess and improve Health-Related Quality of Life (HR-QoL) in trauma patients [2]. Despite the increasing focus on clinical outcomes, more information is still needed to identify those factors that most affect HR-QoL.

The association between injury severity and HR-QoL seems to vary a great deal between studies [3]. Paradoxically, in some studies, the severity of the injury does not seem to correlate with HR-QoL. One explanation that has been suggested is that injury severity scores – which were originally designed to assess trauma mortality – do not necessarily describe how serious the patients feel their injury is [4]. In contrast, some studies have reported that HR-QoL is influenced more by the psychosocial factors of the patient than the severity of the injury [5]. Interestingly, to date, information on whether the two most common scoring systems, the Injury Severity Score (ISS) [6] and the New Injury Severity Score (NISS) [7], have a similar association with HR-QoL has not been thoroughly studied [3].

It seems that the type and anatomical location of the injury may influence post-injury HR-QoL. Of the many types of injuries, spinal cord injuries have been found to have a pronounced negative effect on the HR-QoL of patients [8]. Regarding other risk factors, age or sex do not seem to have much of an effect on HR-QoL, or the results have been inconsistent [9–12]. It can usually be presumed that HR-QoL is reduced after severe injury and remains lower when compared with pre-injury or population reference [3].

The objective of our study is to evaluate HR-QoL after severe injury in Finland and to determine how different injury related factors, such as injury severity scores and injury pattern, and patient-related factors, such as level of education and socioeconomic group, are associated with HR-QoL.

## Material and methods

### Setting

This observational cohort study was conducted at Tampere University Hospital (TAUH), Finland. TAUH serves as a tertiary trauma care unit for the surrounding 3 hospital districts, and it has a catchment area of approximately 900 000 inhabitants. TAUH provides a 24-hour in-house or immediate service in orthopaedic surgery, neurosurgery, anaesthesiology and intensive care, emergency medicine, radiology, internal medicine, plastic surgery, oral and maxillofacial, paediatric, and critical care.

### Patient selection and inclusion/exclusion criteria

**Fig. 1:**
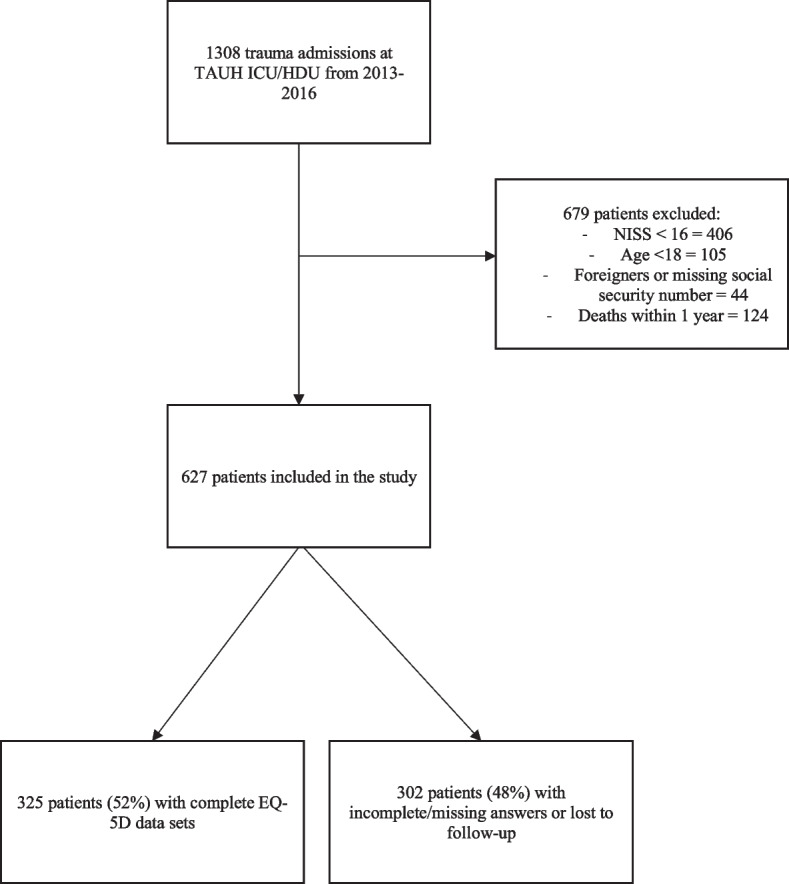
Flowchart of patient selection

All trauma-related admissions (*n*= 1308) with injury-related 10^th^ revision of the International Classification of Diseases and Related Health Problems (ICD-10) codes (S00 – T98) in the Intensive Care Unit (ICU) and the High Dependency Unit (HDU) of TAUH from 2013 through 2016 were retrospectively inspected for patient enrollment. The medical histories, laboratory tests, and radiological examinations of these 1308 patients were reviewed. The following inclusion criteria were used: patient treated at TAUH’s ICU or HDU, aged ≥ 18 years, NISS ≥ 16, alive at 1 year after injury, and possessing a valid Finnish personal identification number to enable comprehensive follow-up. When available, some of the data, i.e., injury severity scores, were extracted from TAUH Trauma Registry, which was founded in 2015. TAUH Trauma Registry’s inclusion criteria match the inclusion criteria of the study. Mortality and cause of death data were obtained from Statistics Finland [13]. A flowchart of the patient enrollment process is presented in Fig. 1

### Injury severity and pattern

All injuries were scored by anatomically-based Abbreviated Injury Scale (AIS) [14] that classifies each injury to nine body regions. To assess injury severity, both the ISS [6] and the NISS [7] were calculated based on the AIS scores. Severe injury was defined as NISS score of at least 16, which is the commonly used definition of severe trauma in studies concerning traumatology [15].

### Evaluation of Health-Related Quality of Life (HR-QoL)

The World Health Organization (WHO) defines Quality of Life as an individual's perception of their position in life in the context of the culture and value systems in which they live and in relation to their goals, expectations, standards, and concerns. Quality of life is, therefore, a broad ranging concept that is affected in a complex way by a person's physical health, psychological state, personal beliefs, social relationships, and their relationship to salient features of their environment [16].

Health-Related Quality of Life (HR-QoL) was evaluated with the EQ-5D-3L [17] questionnaire, which was routinely used in a prospective setting in Tampere University Hospital’s ICU/HDU during the study period. The EQ-5D questionnaire is a 5-dimensioned instrument that assesses mobility, self-care, usual activities, pain/discomfort, and anxiety/depression. Each dimension has 3 levels: no problems, some problems, and extreme problems. EQ-5D is a commonly used instrument in studies assessing HR-QoL after injuries [18]. During their stay in the ICU/HDU, all patients were asked to evaluate their health status prior to admission. If the patient was unable to fill out the forms, the questionnaire was given to the next of kin to complete. After one year, the patients were again asked to re-evaluate their current health status by a study nurse. The EQ-5D results were then converted into a single summary index using a country-specific EQ-5D VAS valuation method. Value sets are normally derived from EQ-5D valuation surveys conducted on a representative sample of the general population of certain country and use scale anchored at 1 = full health and 0 = death. This index value was further compared to the index values of age-matched population norms in Finland [19]. As the index values have not been validated for Finnish patients younger than 25 years, we used the index value for the 25-to-34-year age group (0.909) for the 18-to-25-year age group.

### Education

Information on the education level of each patient was obtained from Statistics Finland [20], which is a national public authority for statistical data. Level of education was defined as low, medium, or high. Those with a low level of education had had at most 9 years of education (elementary school or less), those with a medium level of education had had 11 to 12 years of education (including matriculation examination, vocational qualifications attained in 1-3 years, and further vocational qualifications), and those with a high level of education had had more than 12 years of education (for example, polytechnic degrees, lower and upper-level university degrees, and doctoral education).

### Socioeconomical status

Socioeconomical status refers to an individual’s position in society. The formation of a socioeconomic group for an individual is based on data pertaining to the individual’s main type of activity, occupation, occupational status, and industry. Data for socioeconomical status was obtained from Statistics Finland [21]. Patients were classified into 5 groups: 1. unemployed/unknown, 2. pensioners, 3. students, 4. manual workers, lower-level employees, and self-employed, 5. upper-level employees with administrative, managerial, professional, and related occupations.

### Assessment of physical health

Physical health was assessed by the American Society of Anesthesiologists (ASA) Physical Status Classification System, which is commonly used in ICUs to assess the pre-anesthesia medical co-morbidities of a patient [22].

### Statistics

Statistical analysis was performed with R Studio 4.2.2 (R Foundation for Statistical Computing, Vienna, Austria) with the readxl, ggplot2, dplyr and gridExtra packages. Response variable was HR-QoL index value. Independent variables were grouped into age, sex, ASA, level of education, socioeconomic group, and injury severity scores (NISS, ISS). Injury pattern variables were grouped into head, neck, thorax, abdomen, spine, upper extremity, lower extremity, and unspecified. Minor (AIS 1) injuries, such as cuts and bruises, were excluded from the analysis. Spine injuries with partial or complete injury to the spinal cord (AIS 4 and AIS 5) were additionally assessed. The *x*^2^ test was used for categorical variables. The Mann-Whitney U was used when two groups were compared, and data were not normally distributed. Kruskall-Wallis test was used when more than two groups were compared, and when data were not normally distributed. Spearman's rank correlation coefficient was used to test the correlation between two groups when data were not normally distributed. A *p*-value less than 0.05 was considered statistically significant. Testing of multiple variables may result in false positive (Type I error) equal to the selected significance level, resulting in a higher probability of finding significant results by chance.

## Results

During the study period, a total of 1308 trauma patients were treated at TAUH’s ICU or HDU. Of these, we excluded 406 patients with NISS <16, 105 patients aged <18 years, 124 patients who died within 1 year, and 44 patients who had a missing or altered social security number, i.e., patients who were foreign nationals or patients who had since undergone sex reassignment therapy. In total, 627 patients fulfilled the inclusion criteria and were included in the study. Patient characteristics with differences between responders and non-responders are presented in Table 1. Mean age was 50.4 years (SD 18.4) and 72% of patients were male. Median NISS was 25 (interquartile range 11). Complete data sets with both complete pre- and post-injury EQ-5D questionnaires were available for 325 patients (response rate 52%, 325/627). The responders were older (mean age 53.6 vs. 47.9 years, *p* <0.001), had a higher education level, and were more often employed.

**Table 1:** Demographics of severely injured patients treated at TAUH with NISS‡ > 16 (2013-2016) with comparison between EQ-5D responders and non-responders

	All		Responders		Non-responders		
	*n*= 627		*n*= 325	%	*n*= 302	%	*p*-value
Age, mean (SD)	50.4 (18.4)		53.6 (17.7)		47.0 (18.5)		<0.001a
Sex, female	177	28 %	102	31 %	75	25 %	0.083b
Sex, male	450	72 %	223	69 %	227	75 %	
ASA§							
ASA 1-2	421	67 %	220	68 %	201	67 %	0.83b
ASA 3-4	206	33 %	105	32 %	101	33 %	
Injury severity scores, median (Q1-Q3)							
NISS‡	25 (11)		24 (11)		25 (12)		0.29a
ISS†	17 (9)		17 (11)		17 (9)		0.47a
ICU* days, mean (SD)	3.3 (4.7)		3.5 (5.1)		3.0 (4.1)		0.72a
Education							
Low	169	27 %	84	26 %	85	28 %	0.025b
Middle	319	51 %	154	47 %	165	55 %	
High	139	22 %	87	27 %	52	17 %	
Socioeconomic group							
Unemployed	100	16 %	29	9 %	71	24 %	<0.001b
Pensioners	223	36 %	126	39 %	97	32 %	
Students	26	4 %	13	4 %	13	4 %	
Manual workers, lower-level employees and self-employed	138	22 %	73	22 %	65	22 %	
Upper-level employees with administrative, managerial, professional and related occupations	140	22 %	84	26 %	56	18 %	
Anatomical distribution of injuries, AIS** ≥2							
Head	373	59 %	188	58 %	185	61 %	0.43b
Face	64	10 %	27	8 %	37	12 %	0.13b
Neck	3	0.5%	2	0.6%	1	0.3%	N/A
Thorax	230	37 %	119	37 %	111	37 %	1b
Abdomen	90	14 %	51	16 %	39	13 %	0.38b
Spine	179	29 %	106	33 %	73	24 %	0.024b
with spinal cord injury (AIS** ≥ 4)	66	11 %	42	13 %	24	8 %	0.058b
Upper extremity	147	23 %	80	25 %	67	22 %	0.5331
Lower extremity	153	24 %	77	24 %	76	25 %	0.74b
Unspecified	17	3 %	10	3 %	7	2 %	0.73b

*N/A* n too small for statistical testing

^§^American Society of Anesthesiologist (ASA) physical status classification system

^‡^New Injury Severity Score

^†^Injury Severity Score

^*^ICU = Intensive Care Unit

^**^AIS = Abbreviated Injury Score

a Mann-Whitney U-test

b Chi-square

c Kruskall-Wallis test

Next of kin were reported to have completed the EQ-5D questionnaire in 87/325 cases (26%). There were no differences in change in EQ-5D when at least one of the questionnaires was completed by the next of kin vs. both questionnaires were completed by the patient (-0.119 vs. -0.126, *p*= 0.392). However, when the next of kin returned only the EQ-5D at 12 months, the change in EQ-5D was significantly worse (0.109 vs. 0.272, *p*< 0.001, *n*= 23/325, 7.1%). Patients whose questionnaires was returned by next of kin were more seriously injured (median ISS 17 (IQR 10.75) vs. median 22 (IQR 10), *p*< 0.01, and median NISS 22 (IQR 10) vs. 27 (IQR 10), *p*< 0.001). However, patients age, sex, level of education, were not statically different.

**Fig. 2:**
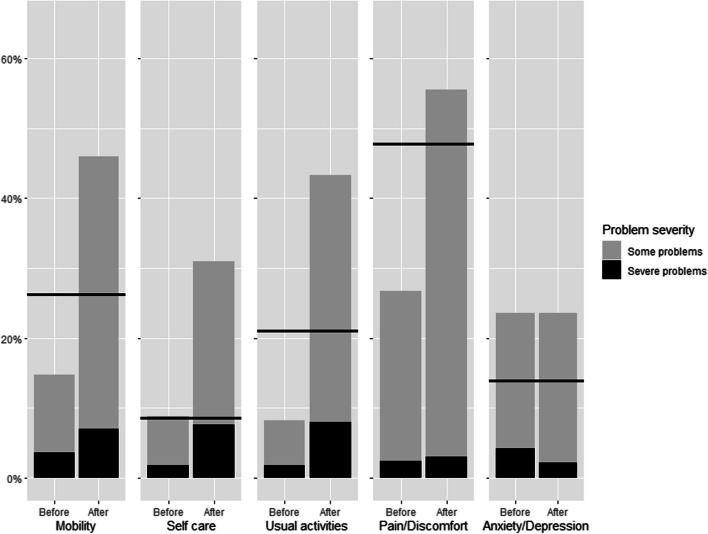
Results (*n*=325) of each EQ-5D dimension with Finnish population norms as a reference (solid line). Y axis represents the rate of problems in percent. The reference line includes both some and severe problems

Compared with Finnish population reference values, the severely injured patients reported more problems in all EQ-5D dimensions except anxiety and depression (Fig. 2). Furthermore, the relative number of reported problems after injury exceeded the relative number of reported problems of the reference population in all five EQ-5D dimensions. Comparison of pre- and post-injury HR-QoL to age-specific population norms of Finland in presented in Table 2. Pre-injury HR-QoL exceeded population norms in patients aged 65 and over. When compared to population norm post-injury HR-QoL was lowest in patients aged 25-34 years and exceeded the population norm in the patients aged > 75 years.

**Table 2:** Pre- and post-traumatic Health-Related Quality of Life (HR-QoL) compared to age-specific population norms of Finland, *n*= 325

	*n*=325	%	Pre-injury - population norm, mean (sd)		Post-injury HR-QoL - population norm, mean (SD)	
Age				*p*-value		*p*-value
18-24	28	9 %	0.0350 (0.0851)	<0.001*	-0.0696 (0.204)	<0.001*
25-34	37	11 %	0.00243 (0.137)		-0.146 (0.249)	
35-44	29	9 %	0.0222 (0.125)		-0.0600 (0.175)	
45-54	56	17 %	0.0507 (0.191)		-0.0567 (0.201)	
55-64	72	22 %	0.0548 (0.200)		-0.0276 (0.248)	
65-74	69	21 %	0.168 (0.184)		-0.0167 (0.273)	
> 75	34	11 %	0.277 (0.199)		0.166 (0.172)	

^*^Kruskall-Wallis test

The average decrease between pre- and post-injury HR-QoL was -0.121 (SD 0.230). Associations between EQ-5D values injury- and patient-related factors are presented in Tables 3 and 4, respectively**.** The severity of an injury did not have an effect on the decrease in HR-QoL. Length of stay at the ICU had a weak negative correlation with post-injury HR-QoL and weak positive correlation with the change in HR-QoL. The effect of ICU stay is presented in days. The largest mean decrease in HR-QoL was in patients with spinal cord injury (Spine AIS ≥ 4) (-0.338 (SD 0.136)), spine injury in general (Spine AIS ≥ 2 (-0.201 (SD 0.279)), and those who had lower education (-0.157 (SD 0.231)). Patients with spinal cord injury (Spine AIS ≥ 4) reported the lowest post-injury HR-QoL (0.547 (SD 0.270)).

**Table 3:** Pre- and post-traumatic Health-Related Quality of Life (HR-QoL) with injury-related factors. Patients with complete EQ-5D data sets, *n*= 325. Statistical testing was done between group differences for pre-injury and post-injury variables, and between pre-injury and post-injury values (change)

		*n*=325	%	Pre-injury HR-QoL, mean (SD)		Post-injury HR-QoL at 12 months, mean (SD)		Change in HR-QoL, mean (SD)	*p*-value
Injury Severity Scores									
NISS^b^					*p*-value		*p*-value		*p*-value
	16-24	170	52 %	0.904 (0.168)	0.039*	0.783 (0.220)	0.29*	-0.121 (0.229)	0.91*
	25-39	124	38 %	0.870 (0.184)		0.754 (0.229)		-0.117 (0.238)	
	40-75	31	10 %	0.843 (0.189)		0.708 (0.288)		-0.135 (0.221)	
ISS^a^									
	9-15	83	26 %	0.918 (0.166)	0.013*	0.843 (0.177)	0.0012*	-0.0749 (0.178)	0.14*
	16-24	140	43 %	0.887 (0.175)		0.753(0.231)		-0.133 (0.246)	
	> 25	102	31 %	0.858 (0.185)		0.716 (0.253)		-0.141 (0.244)	
Injury pattern (AIS^c^ ≥2)									
Head									
	Yes	188	58 %	0.864 (0.190)	0.007**	0.783 (0.208)	0.24**	-0.0809 (0.207)	0.002**
	No	137	42 %	0.915 (0.153)		0.739 (0.257)		-0.176 (0.251)	
Face									
	Yes	27	8 %	0.902 (0.165)	0.55**	0.797 (0.215)	0.46**	-0.105 (0.144)	0.79**
	No	298	92 %	0.884 (0.178)		0.762 (0.232)		-0.122 (0.238)	
Neck									
	Yes	2	0.6%	0.652 (0.0686)	0.034**	0.553 (0.232)	0.17**	-0.0995 (0.301)	0.85**
	No	323	99 %	0.887 (0.176)		0.766 (0.231)		-0.121 (0.231)	
Thorax									
	Yes	119	37 %	0.913 (0.144)	0.061**	0.780 (0.220)	0.54**	-0.133 (0.199)	0.43**
	No	206	63 %	0.870 (0.192)		0.756 (0.237)		-0.114 (0.248)	
Abdomen									
	Yes	51	16 %	0.928 (0.110)	0.16**	0.779 (0.203)	0.99**	-0.149 (0.194)	0.21**
	No	274	84 %	0.878 (0.186)		0.762 (0.236)		-0.116 (0.237)	
Spine									
	Yes	106	33 %	0.884 (0.165)	0.37**	0.684 (0.257)	<0.001**	-0.201 (0.279)	<0.001**
	No	219	67 %	0.886 (0.183)		0.804 (0.207)		-0.0822 (0.193)	
with spinal cord injury (AIS** ≥ 4)									
	Yes	42	13 %	0.885(0.175)	0.71**	0.547(0.270)	<0.001**	-0.338 (0.314)	<0.001**
	No	283	87 %	0.886(0.177)		0.797(0.206)		-0.0886 (0.197)	
Upper extremity									
	Yes	80	25 %	0.919 (0.143)	0.055**	0.823 (0.178)	0.032**	-0.0964 (0.136)	0.48**
	No	245	75 %	0.875 (0.186)		0.746 (0.243)		-0.129 (0.254)	
Lower extremity									
	Yes	77	24 %	0.915 (0.129)	0.30**	0.785 (0.192)	0.83**	-0.130 (0.178)	0.40**
	No	248	76 %	0.876 (0.189)		0.759 (0.242)		-0.118 (0.245)	
Unspecified									
	Yes	10	3 %	0.823 (0.215)	0.21**	0.723 (0.238)	0.51**	-0.100 (0.201)	0.82**
	No	315	97 %	0.888 (0.176)		0.766 (0.231)		-0.122 (0.232)	

^a^Injury Severity Score

^b^New Injury Severity Score

^c^AIS = Abbreviated Injury Score

^*^Kruskall-Wallis test

^**^Mann-Whitney U-test

**Table 4:** Pre- and post-traumatic Health-Related Quality of Life (HR-QoL) with patient-related factors. Patients with complete EQ-5D data sets, *n*= 325. Statistical testing was done between group differences for pre-injury and post-injury variables, and between pre-injury and post-injury values (change)

	*n*=325	%	Pre-injury value, mean (SD)		Post-injury HR-QoL at 12 months, mean (SD)		Change, mean (SD)	*p*-value
Age				*p*-value		*p*-value		*p*-value
18-24	28	9 %	0.944 (0.0851)	0.042*	0.839 (0.204)	0.37*	-0.105 (0.173)	0.51*
25-34	37	11 %	0.911(0.137)		0.763 (0.249)		-0.148 (0.256)	
35-44	29	9 %	0.900 (0.125)		0.818 (0.175)		-0.0821 (0.138)	
45-54	56	17 %	0.886 (0.191)		0.778 (0.201)		-0.107 (0.180)	
55-64	72	22 %	0.836 (0.200)		0.753 (0.248)		-0.0824 (0.240)	
65-74	69	21 %	0.906 (0.184)		0.721 (0.273)		-0.185 (0.292)	
> 75	34	11 %	0.860 (0.199)		0.749 (0.172)		-0.110 (0.208)	
Sex, female	102	31 %	0.846 (0.197)	<0.001**	0.752 (0.236)	0.50**	-0.0934 (0.249)	0.19**
Sex, male	223	69 %	0.904 (0.164)		0.770 (0.229)		-0.133 (0.222)	
ASA^a^ 1-2	220	68 %	0.928 (0.126)	<0.001**	0.803 (0.210)	<0.001**	-0.125 (0.210)	0.87**
ASA^a^ 3-4	105	32 %	0.797 (0.229)		0.685 (0.253)		-0.112 (0.272)	
ICU Days mean (SD)	3.5 (5.1)		-0.068 ***	0.21	-0.20 ***	<0.001	-0.20 ***	<0.001
Level of education								
Low	84	26 %	0.896 (0.161)	0.52*	0.739 (0.206)	0.054*	-0.157 (0.231)	0.022*
Middle	154	47 %	0.873 (0.187)		0.754 (0.251)		-0.119 (0.243)	
High	87	27 %	0.898 (0.172)		0.809 (0.211)		-0.0897 (0.206)	
Social economic group								
Unemployed	29	9 %	0.826 (0.207)	<0.001*	0.719 (0.259)	<0.001*	-0.108 (0.269)	0.23*
Pensioners	126	39 %	0.847 (0.210)		0.701 0.248)		-0.146 (0.273)	
Students	13	4 %	0.905 (0.109)		0.784 (0.155)		-0.121 (0.130)	
Manual workers, lower-level employees and self-employed	73	22 %	0.933 (0.120)		0.806 (0.184)		-0.127 (0.183)	
Upper-level employees with administrative, managerial, professional, and related occupations	84	26 %	0.920 (0.142)		0.838 (0.214)		-0.0827 (0.195)	

^a^American Society of Anesthesiologist (ASA) physical status classification system

^*^Kruskall-Wallis test

^**^Mann-Whitney U-test

^***^Spearman's rank correlation coefficient

Pre-injury HR-QoL was lower in patients who were older, female, had worse previous physical health, were unemployed, had higher injury severity scores, and had sustained injuries to head or neck. Neck injuries were observed in only two patients who had previously reported problems in self-care, and anxiety and depression. Post-injury HR-QoL was lower in patients with worse physical health, were unemployed or pensioners, or had injuries to the spine.

## Discussion

In this study, we investigated the HR-QoL of seriously injured patients and determined the associations between various patient- and injury-specific factors and HR-QOL. As previously reported in the literature [3, 23], the impact of a serious injury on the patient's HR-QoL is significant and HR-QoL after injury remains lower compared to the population reference values. In the present study, however, the severity of the injury (measured by ISS and NISS) had no significant association with the decrease in HR-QoL. Among the different types of injuries, spine and spinal cord injuries in particular had a negative impact on HR-QoL, whereas the impact of head injuries on HR-QoL appeared to be less. A longer stay in intensive care seems to be associated with lower HR-QoL. In addition, a low level of education also seems to be associated with lower HR-QoL.

In the literature, the relationship between injury severity scores and HR-QoL varies [3, 18]. Contrary to what one might expect, higher injury severity scores have not always led to lower quality of life [3]. It is likely, therefore, that the scoring systems (i.e., ISS, NISS), which were originally created to predict the mortality of trauma patients, are not suitable for measuring HR-QoL. For example, although a life-threatening hemorrhage in the abdomen and a fracture of the spine can produce the same score, recovery from these injuries may be completely different. Indeed, it is likely that the person injured in the abdominal area may have very few symptoms or be even asymptomatic after a year has passed, whereas the person with a spinal fracture may still need significant help with their daily activities. For this reason, the clinician should not use injury severity scores alone to evaluate a patient's future HR-QoL. Instead, the expected consequences of the injury should first be considered. The differences in how the scores are calculated may also produce heterogeneity in the existing literature. For example, although both ISS and NISS scores are derived from the AIS scoring system, the ISS adds only the most severe body region, while the NISS can add up to three injuries per body region. Therefore, an injury to the head with a skull fracture, subdural hemorrhage, and contusion will provide significantly different severity scores, depending on whether it was evaluated by the ISS or NISS. However, in the current study, neither the ISS nor the NISS appeared to be good predictors of HR-QoL.

Regarding the types of injuries, our study is in the line with earlier reports that injuries to the spine and especially to the spinal cord have the worst negative effect on HR-QoL [8]. Spinal cord injuries are often associated with many kinds of permanent disabilities, and the consequences for the patient are often life changing. Chronic pain is common and up to 77% of patients with spinal cord injury report chronic pain [24]. The level of spinal cord injury also matters, and patients with tetraplegia frequently report more problems with physical functioning and bodily pain than patients with paraplegia [25]. Patients who sustained head injuries reported lower pre-injury HR-QoL but eventually made a better recovery than patients without head injuries. Compared to other trauma patients, patients with mild head injury have been found to report more problems with mental health prior to injury. Although this may explain the worse baseline situation, it does not prevent the patients' mental health from improving later [26]. The number of physical symptoms caused by a head injury has also been found to decrease over time, [27, 28], although the post-injury HR-QoL of patients with head injury generally remains reduced compared to population norms [11].

A low level of education was associated with a larger decrease in HR-QoL. This finding is in line with a study by Haider who noted that although information on level of education is not routinely collected in trauma registries, a low level of education is the most predictive variable of worse long-term outcome after severe trauma [29]. Although no differences were observed in pre- or post-injury HR-QoL, we noticed that the higher the patient's education level, the smaller the subsequent decrease in HR-QoL. It may be, therefore, that a high level of education acts as a protective factor. In addition to physical qualities, recovering from a serious injury requires that patients have good mental skills, i.e., resilience, coping skills, and self-efficacy [24, 25, 29], all of which are likely to be greater in patients who are more highly educated. Returning to working life is also a recognized factor for quality of life [5, 28]. It maybe that returning to working life after injury is more successful for a better educated person than, for example, for a less educated person whose work profile is likely to be more physically demanding.

Low socioeconomic status has previously been found to be linked to a reduction in HR-QoL [18]. The perceived HR-QoL was the lowest among the unemployed and older populations, which may well be due to a previous physical or mental illness, such as depression or anxiety symptoms. In our data, however, we found no differences in HR-QoL between different socioeconomic groups during recovery from injury. This finding may be influenced by the current practice in Finland that all severe injuries are treated under universal health care, where the patient's wealth or income level does not affect where the patient is treated and what the treatment includes. It should be noted that the definition of socioeconomic status or group varies between different studies and can be based on, for example, current annual income or residential area [30]. In our study, data on occupation were used for definition purposes. Although a different job title does not necessarily directly mean a higher socioeconomic status, it is reasonable to presume that a working person with perhaps a higher education has better access to economic resources and social position in relation to others than an unemployed person or pensioner. The effects of having a higher socioeconomic status are likely to be similar to those of having a higher level of education, which are linked in many ways in Finland [31].

Younger patients perceived their pre-injury HR-QoL to be better than older patients, which is a common finding found in many population-level studies. An exception was made by patients older than 65 years whose pre-injury HR-QoL even exceeded the population-level reference values. It is possible that this may have been caused by recall bias. However, it may be that the patients in our study were, in general, more active than older people in previous studies, or they had perhaps adapted better to previous minor ailments. Like in some previous studies, the change in HR-QoL between different age groups was not statistically different, suggesting equal recovery regardless of patient age [9, 11, 12].

### Weaknesses

As in many studies on HR-QoL, various psychological biases (i.e., recall bias) can affect the results. Although the HR-QoL data were prospectively collected as soon as possible in the ICU, it may still be that some patients rate their pre-injury HR-QoL to be better or worse than it was [32, 33]. However, as data collection started early in the ICU/HDU, the risk for these biases is likely reduced. Even though the patients were contacted by phone, the response rate remained moderate. When evaluating the response rate, it should be borne in mind that the study cohort consisted of only seriously injured patients. After a severe injury, the first-year mortality rate is 10% to 15%, and the consequences of the injury can act as a barrier to reaching the patient. It may be, for example, that a patient has been subjected to long-term institutional care, has no family members that could have answered the phone, or has simply lost interest in the study. It should be noted, however, that the response rate in our study does not differ from most other HR-QoL studies, especially when the data consist of seriously injured patients. There were some differences between responders and non-responders: the responders were older (mean age 53.5 vs. 47.0 years, *p*<0.001), had a higher level of education, were of a higher socioeconomic group, and the rate of spine injuries was higher (33% vs. 24%, *p*=0.024). We believe, therefore, that the study still provides a reliable picture of these patients. It should be noted that in present study multiple variables were tested for statistical significancy. Therefore, the possibility that significant findings occur by change is increased. However, we argue that all the tests were planned in advance and all variables had sound reason to be tested for significancy due to known associations from earlier studies.

### Strengths

The definitive strength of this study is the study population which consists of all severely injured trauma patients treated at single major trauma center responsible for all major trauma in its catchment area during the study period. The data on HR-QoL were collected prospectively and the data collection on HR-QoL started at the earliest possible time in the ICU/HDU, which is likely to have reduced the risk for recall bias. To complement the analysis, additional information on injury profile and diagnostics test results were added retrospectively from the electronic medical records.

## Conclusions

After a serious injury, many patients have permanent disabilities which reduce HR-QoL. Injury scoring systems intended for assessing the risk for death did not seem to associate with HR-QoL and are not, therefore, a meaningful way to predict the future HR-QoL of a severely injured patient. There were some differences between the different injury types. For example, the decrease in HR-QoL is particularly associated with an injury to the spine or spinal cord, whereas the average recovery from head injuries seems to be better. Patients of different ages appear to regain HR-QoL similarly. Although there were significant differences in baseline HR-QoL levels between the different socioeconomic groups, recovery from injuries appears to be similar, which is likely due to equal access to high quality trauma care provided by universal health care. A low level of education was, however, associated with worse recovery.

## Data Availability

The data that support the findings of this study are not openly available due to privacy restrictions but are available from the corresponding author (AR) upon reasonable request.

## Abbreviations

AIS: Abbreviated Injury Scale
ASA: American Society of Anesthesiologists (ASA) Physical Status Classification System,
HDU: High Dependency Unit
HR-QoL: Health-Related Quality of Life
ICU: Intensive Care Unit
ISS: Injury Severity Score
LOS: Length of Stay
MAIS: Maximum Abbreviated Injury Score
NISS: New Injury Severity Score
TAUH: Tampere University Hospital
